# Identification of Reliable Target Brain Regions for Enhancing Object–Location Memory by Brain Stimulation

**DOI:** 10.1002/brb3.70658

**Published:** 2025-07-09

**Authors:** Mohamed Abdelmotaleb, Filip Niemann, Harun Kocataş, Leonardo M. Caisachana Guevara, Alireza Shahbabaie, Robert Malinowski, Steffen Riemann, Anna Elisabeth Fromm, Dayana Hayek, Daria Antonenko, Marcus Meinzer, Agnes Flöel

**Affiliations:** ^1^ Department of Neurology University Medicine Greifswald Greifswald Germany; ^2^ Department of Psychology Greifswald University Greifswald Germany; ^3^ German Center for Neurodegenerative Diseases (DZNE Site Greifswald) Greifswald Germany

**Keywords:** object–location memory (OLM), test–retest reliability (TRR), transcranial direct current stimulation (tDCS), functional magnetic resonance imaging (fMRI)

## Abstract

**Introduction:**

Object–location memory (OLM) is essential for remembering the locations of objects within an environment and is often impaired in aging and neurodegenerative diseases. Transcranial direct current stimulation (tDCS) has shown promise for improving OLM, although study outcomes have varied considerably. This study aimed to identify key brain regions involved in OLM that may serve as stimulation targets for future tDCS research and to assess the test–retest reliability (TRR) of both behavioral and functional magnetic resonance imaging (fMRI) data.

**Methods:**

Twenty healthy young adults (10 females; mean age = 25.0 years, standard deviation [SD] = 5.56) completed two task‐based fMRI sessions using parallel versions of an OLM task. Participants learned associations between house images and their locations on a two‐dimensional street map across four feedback‐based learning stages. Sham tDCS was administered in both sessions using a focal 3 × 1 electrode montage. TRR was assessed using intraclass correlation coefficients (ICCs) for behavioral performance and task‐related fMRI activation.

**Results:**

Behavioral data showed significant improvements in response accuracy (estimate = 0.211, *p* < 0.001) and a reduction in response latency (estimate = −0.050, *p* < 0.001) across learning stages. fMRI analysis revealed predominantly right‐lateralized activation, including the right hippocampus, the fusiform gyrus, the precuneus, and the lateral temporo‐occipital areas. Behavioral measures showed moderate to good TRR (accuracy ICC = 0.801; reaction time ICC = 0.705). Task‐related fMRI activity demonstrated good‐to‐excellent TRR in key regions, including the fusiform and temporo‐occipital cortices.

**Conclusion:**

These findings support the validity of our OLM paradigm for assessing brain stimulation effects and highlight potential cortical targets for future tDCS interventions. The observed reliability of behavioral and neural measures further reinforces the utility of this protocol in crossover study designs.

## Graphical abstract

Using fMRI during an object–location memory task, we identified right‐lateralized cortical regions—lateral occipital–temporal cortices, fusiform, and precuneus—as viable brain stimulation targets. Robust behavioral gains and test–retest reliability endorse the paradigm's potential for future brain stimulation research.

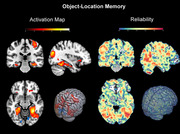

## 1 Introduction

Object–location memory (OLM), a subtype of spatial memory, is essential for remembering the locations of objects within dynamic environments (Postma et al. 2004). It is crucial for daily activities, such as locating personal belongings or navigating unfamiliar surroundings (Postma et al. 2008). OLM shows a noticeable decline with normal aging, a process that is exacerbated in neurodegenerative diseases (Kessels et al. 2007, 2010; Ossher et al. 2013).

Given its high ecological relevance, several studies have attempted to enhance OLM through training, transcranial direct current stimulation (tDCS), or a combination of both (Flöel et al. 2012; England et al. 2015; Antonenko et al. 2018; Bjekić et al. 2019; de Sousa et al. 2020; Jones et al. 2021; Fromm et al. 2024). As a non‐invasive brain stimulation technique, tDCS modulates neural excitability and plasticity in target brain regions associated with specific cognitive processes (Nitsche and Paulus 2000). However, tDCS studies on OLM in healthy young and older adults, as well as individuals with mild cognitive impairment, have yielded mixed results. Although some studies reported immediate or delayed performance benefits (Flöel et al. 2012; Prehn et al. 2017; Antonenko et al. 2018; Bjekić et al. 2019), others found no improvement (Külzow et al. 2017; Fromm et al. 2024).

Notably, previous tDCS research has devoted little attention to the validity of the target regions for tDCS in specific OLM paradigms or populations, which might have contributed to these variable outcomes. The key hub for object–location binding (i.e., the hippocampus) cannot be accessed directly by tDCS. On the basis of an influential model proposed by Postma et al. (2008), the majority of previous tDCS studies have targeted the right temporo‐parietal cortex (Flöel et al. 2012; England et al. 2015; Külzow et al. 2017; Antonenko et al. 2018; Bjekić et al. 2019; Fromm et al. 2024). However, functional neuroimaging studies of OLM have revealed several other accessible cortical target regions for brain stimulation, including prefrontal, parietal, or lateral temporo‐occipital regions (Owen et al. 1996; Petersson et al. 2001; Slotnick et al. 2003; Hales and Brewer 2013; Gillis et al. 2016). Moreover, studies employing a range of brain mapping methods—including functional magnetic resonance imaging (fMRI), PET, and lesion‐based approaches—have reported variable networks for OLM‐related activity (Petersson et al. 2001; Ross and Slotnick 2008; Zimmermann and Eschen 2017), highlighting the complexity of this memory function and the need to optimize tDCS target selection based on the specific paradigm and population under study.

In addition, crossover or longitudinal tDCS studies need to ensure the stability of behavioral and/or neural outcomes used to investigate the effects of brain stimulation interventions (Meinzer et al. 2024). This is particularly important given that both behavioral and task‐based functional imaging outcomes often have low test–retest reliability (TRR) (Smith et al. 2005; M. L. Elliott, Knodt, et al. 2020; Judd et al. 2024), which may contribute to the variability observed in tDCS studies (Madhavan et al. 2016). This highlights the need to investigate the reproducibility of behavioral and neural responses elicited by specific paradigms and in the target population before implementation in tDCS studies, especially for novel experimental paradigms.

The present study addressed these challenges by employing a novel fMRI adaptation of an established OLM paradigm (Flöel et al. 2012; Külzow et al. 2014), designed for use in a future crossover study examining the behavioral and neural effects of tDCS. The first aim of this study was to identify potential target regions for tDCS using this OLM paradigm. The second aim was to formally assess the TRR of task performance and functional imaging correlates across two testing sessions. Both served to ensure the suitability of the paradigm for investigating tDCS effects in a subsequent sham tDCS–controlled intra‐scanner crossover study.

## 2 Materials and Methods

This study describes the results of the preparatory phase of a larger, multicenter, double‐blinded, sham‐controlled crossover tDCS study aimed at investigating the behavioral and neural effects of tDCS on motor and cognitive functions across multiple functional domains (https://www.memoslap.de/de/forschung/). The primary objectives of this preparatory phase are to validate and assess the reliability of paradigms developed for specific functional domains (i.e., visual‐spatial, language, executive, and motor functions) and to determine potential tDCS targets for subsequent phases. This study was pre‐registered on the Open Science Framework (OSF). The pre‐registration protocol is available under OSF Registries (https://osf.io/t37u2).

### 2.1 Experimental Design

#### 2.1.1 Participants and Study Overview

Twenty healthy young‐ to middle‐aged adults (range 18–45) participated (10 females; mean age = 25; standard deviation [SD] = 5.56). All participants were right‐handed, as evaluated by the Edinburgh Handedness Inventory (Oldfield 1971); they were native German speakers and reported no history of neurological or psychiatric conditions. Participants also had no history of alcoholism, drug use, or contraindications for MRI or tDCS (Antal et al. 2017).

#### 2.1.2 Baseline Assessments

Participants underwent an initial telephone screening to confirm their eligibility. Following this, a comprehensive neuropsychological assessment was conducted to ensure normal cognitive function. Participants were also screened for depression (German version of Beck's Depression Inventory, BDI‐II; Kühner et al. 2007).

During the neuropsychological assessment, verbal memory was assessed with the German Auditory Verbal Learning Test (verbal learning and memory test [VLMT]; Müller et al. 1997). Visual–spatial memory was examined using the Rey–Osterrieth Complex Figure Test (ROCFT; Zhang et al. 2021). Short‐term memory was assessed with the Digit Span (Woods et al. 2011). Executive functioning was assessed using the Trail Making Test (Parts A and B) (Tombaugh 2004), the Stroop Color–Word Test (Van der Elst et al. 2006), and the Regensburg Verbal Fluency Test (Harth and Müller 2004). Verbal intelligence and vocabulary knowledge were assessed with the German version of the multiple‐choice vocabulary test (MWT; Lehrl 2018).

Participants also completed a short version of the OLM task, both as practice for the main intra‐scanner task and to classify them as “high” or “low” performers in the learning task. This classification is relevant to tDCS outcomes (Meinzer et al. 2013; Perceval et al. 2020) and can serve as a predictor of effects in subsequent active stimulation phases of the larger study. Participants’ characteristics are provided in Table 1.

**TABLE 1:** Descriptive statistics for demographic, neuropsychological, and experimental behavioral performances for the study sample.

		Mean (SD)
Age [years]		25.05 (5.56)
Education [years]		15.9 (3.01)
Beck Depression Inventory (BDI‐II)		5.2 (4.76)
Verbal Learning Memory (VLMT) [%]		81.66 (14.4)
Spatial memory (RCFT) [%]		78.88 (12.02)
Digit Span Test [*n*]	Digit forward Digit Backward	7.9 (2.17) 8.15 (1.98)
Trail Making Test (TMT) [s]	A B	22.75 (4.94) 44.20 (12.82)
Stroop Test [s]	Words Color Interference	27.55 (3.26) 39.20 (4.61) 58.45 (6.77)
Regensburg Word Fluency Test (RWT) [*n*]	Phonemic Semantic Switch	19.55 (5.42) 31.05 (7.62) 19.05 (3.03)
Multiple‐Choice Vocabulary Test (MWT) [*n*]		27.45 (2.56)
Short version OLM [%]		79 (19)

*Note*: Descriptive statistics are presented as mean values with standard deviations (SD) in parentheses. Age and education are reported in years. The Beck Depression Inventory (BDI‐II) measures depressive symptoms. The Verbal Learning and Memory Test (VLMT) and the Rey–Osterrieth Complex Figure Test (RCFT) assess verbal and spatial memory performance, respectively, as percentages. The Digit Span Test evaluates short‐term and working memory through digit forward and backward recall (*n* = number of correct sequences). The Trail Making Test (TMT) measures working memory by recording the times (in seconds) for parts A and B. The Stroop Test assesses executive functioning with times recorded for word reading, color naming, and interference conditions. The Regensburg Word Fluency Test (RWT) includes phonemic, semantic, and switching fluency (*n* = number of words). The Multiple‐Choice Vocabulary Test (MWT) measures vocabulary knowledge, and the Short Version Object‐Location Memory (OLM) assesses the mean of spatial memory performance in percentage form.

#### 2.1.3 OLM Task Design

Each participant underwent two task fMRI sessions, separated by at least 1 week. During each session, participants performed one of two parallel versions of the OLM task, adapted from previous studies by our group (Flöel et al. 2012; Prehn et al. 2017; Antonenko et al. 2018; de Sousa et al. 2020). In brief, the paradigm involves learning associations between objects (houses on a two‐dimensional [2D] map) and their locations through repeated presentations, utilizing both instruction‐ and feedback‐based mechanisms. The task employed in the present study uses a block design with four learning stages, which minimizes the effects of confounding factors related to time on task, novelty of being in the scanner, and fatigue (Sliwinska et al. 2017).

**FIGURE 1:**
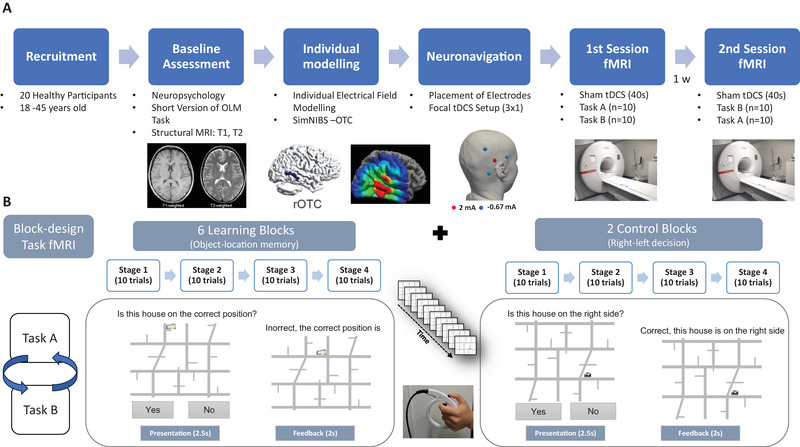
**Overview of the experimental design and object location memory (OLM) task**. (A) Flowchart of the experimental procedure. Twenty healthy participants (aged 18–45) underwent baseline assessments, including neuropsychological testing, a short version of the OLM task, and structural MRI (T1 and T2). Individualized electric field modeling was performed using SimNIBS, targeting the right occipito‐temporal cortex (ROTC), followed by neuronavigation‐guided focal transcranial direct current stimulation (tDCS) (3 × 1 montage). Participants completed two task‐based fMRI sessions, separated by 1 week. All participants performed both Task A and Task B, with the task order counterbalanced across sessions (i.e., 10 participants completed Task A in Session 1 and Task B in Session 2, whereas the other 10 participants completed the tasks in the reverse order). Each session included brief sham tDCS and task performance inside the scanner. (B) Block design of the fMRI task. Each task session consisted of six learning blocks (OLM task) and two control blocks (right‐left decision). Each block consisted of four stages (10 trials per stage). In the OLM task, participants judged whether a house was in the correct location and received feedback. In the control task, they judged whether a house was located on the right or left side of the map. Each trial consisted of a 2.5‐s stimulus presentation followed by 2 s of feedback. Responses were made using MRI‐compatible response grips. fMRI, functional magnetic resonance imaging.

An overview of the intra‐scanner task design and the experimental procedures is provided in Figure 1. In the learning task, participants were required to indicate whether a house was correctly positioned on a schematic street map, receiving immediate feedback to assist in learning the correct locations of each house across repeated trials. Without prior training in the novel houses and location combinations, participants were asked to judge whether the house they saw was at the “correct” position on a screen. Following each trial, they received written feedback indicating whether their response had been correct or incorrect; regardless of their accuracy, this feedback also provided the correct location of the house. If participants failed to respond within the allotted time, they were informed that they had missed the trial but received the correct house's location information. When a house was presented for the first time, participants were encouraged to guess whether its association with the map's shown position was accurate or not, utilizing the subsequent feedback to learn about the correct position. A control task required determining whether a house was on the left or right side of the map displayed on the screen, controlling for perceptual and motor demands without involving memory associations.

Each learning block comprised 40 trials, repeated across six blocks (30 object–location associations total). In each stage, five houses were presented pseudorandomly in correct and incorrect locations (five of each). The same set of five houses was repeated across all four stages of a block, enabling participants to learn five unique object–location associations per block. The control task followed an identical structure, consisting of two blocks of four stages each, and utilized a total of 10 different houses for right/left decisions.

In both experimental conditions, the trial duration was 4.5 s (Figure 1). Each trial presented a house positioned at a specific location on the map, and the same house picture was never presented consecutively. The house–location association was presented for 2.5 s, and participants were required to respond during this time frame. An additional 2 s were included to provide trial‐specific feedback. Therefore, participants had to provide their response as quickly and accurately as possible by pressing the response buttons of an MRI‐compatible response grip with their left hand's index finger or thumb for “yes” and “no” responses, respectively. Participants were informed that only their first response was recorded to ensure a balance between accuracy and response latency.

A fixation cross was incorporated during the experiments to establish a low‐level baseline condition. Each fixation cross was synchronized with the experimental trial duration (4.5 s) and was presented after each stage (10 trials) within each learning block. Additionally, after each learning block, a longer fixation cross lasting 18 s was introduced to facilitate the transition from the learning to the control task or the reverse. The order of the learning and control blocks was randomly assigned by the presentation software. At the beginning of each block, participants received a brief written speech bubble to introduce the respective task conditions.

Participants were shown instructions for performing the task before their first session. Task order was randomized, and two counterbalanced task versions (A and B) featured unique houses and rotated maps to maintain comparable difficulty and performance across sessions. Stimuli included 90 images of real‐world buildings selected for recognizability and complexity (Flöel et al. 2012), paired with schematic street maps. Maps were divided into quadrants with assigned correct and incorrect locations, ensuring balanced and randomized placement over the entire street map across trials.

Stimuli were presented using Presentation software (Version 20.1, Neurobehavioral Systems Inc., Berkeley, CA, USA) and displayed via an MRI‐compatible screen and mirrors. MRI‐compatible response grips were used for behavioral decisions (NordicNeuro, Norway, www.nordicneurolab.com).

#### 2.1.4 Focal Sham tDCS

The present study aimed to identify key brain regions activated during fMRI using a specific OLM paradigm. In addition, it aimed to assess the paradigm's TRR. Sham tDCS was administered during both imaging sessions to establish a reliable and feasible workflow for conducting tDCS‐fMRI experiments, including neuronavigated electrode placement, intra‐scanner tDCS procedures, task implementation during scanning, and optimization of fMRI acquisition parameters. Furthermore, the use of sham stimulation provided a methodologically comparable control condition for future phases of an overarching project that will involve active stimulation (Meinzer et al. 2024).

A focal 3 × 1 tDCS setup with a multi‐channel direct current (DC) stimulator was used. Sham tDCS was administered during functional imaging sessions using MRI‐compatible equipment, including a multi‐channel DC stimulator (DC‐STIMULATOR MC, NeuroConn GmbH, Germany), circular conductive rubber electrodes (diameter = 2 cm), and cables (for details, see Niemann et al. 2024). The sham stimulation protocol comprised 10‐s ramp‐up and ramp‐down periods, with 20 s of DC application at 2 mA in between. This protocol is designed to mimic the initial physical sensation typically associated with active tDCS without inducing sustained alterations in cortical excitability (Nitsche et al. 2008). Sham tDCS was administered using the same montage and current intensity that will be used in the subsequent active and sham tDCS arms of the overarching project.

The right lateral occipito‐temporal cortex (right occipito‐temporal cortex [ROTC]) was chosen on the basis of a previous neuroimaging study (Gillis et al. 2016) to serve as a preliminary target at this preparatory stage. Recent evidence demonstrated structural and functional connectivity between the lateral occipital and temporal cortices and the hippocampus (Dalton et al. 2022; Rolls et al. 2024). Although the hippocampus is known to play a central role in OLM, it is not directly accessible by tDCS. Therefore, targeting functionally connected neocortical regions such as the ROTC may provide an effective, indirect approach to modulate hippocampal activity (Nilakantan et al. 2017; Tambini et al. 2018).

The anode was placed over the individually localized target region and was surrounded by three equally spaced cathodes (the distance between cathodes was kept constant between participants). The distance of the cathodes was determined by a simulation pilot study, which ensured that this setup would reach an intended threshold of stimulation intensity of 0.2 mA across participants and projects of the Research Unit MeMoSLAP. Electrode positioning was standardized using a 3D‐printed thermoplastic spacer with a 3 cm radius (center‐to‐center distance between the anode and cathodes, Niemann et al. 2024).

The algorithm used for montage individualization aimed to ensure that the fields are robustly centered on the individual target and minimize co‐stimulation of surrounding areas. Tissue segmentation of the head and brain (based on individual tetrahedral head meshes generated from T1‐ and T2‐weighted images using “charm”) and electric field (EF) modeling was performed using SimNIBS v4 (simnibs.org; Thielscher et al. 2011; Puonti et al. 2020).

This approach ensured precise electrode placement during the fMRI‐tDCS sessions. Although this study employed only sham tDCS to investigate potential target regions, these measures were implemented to simulate the planned, individualized modeling for the subsequent active stimulation phases of the larger tDCS study.

#### 2.1.5 MRI Acquisition

MRI data were acquired at a 3.0 Tesla Siemens MAGNETOM Vida scanner (Siemens Healthineers, Germany) at the University Medicine Greifswald with a 64‐channel head–neck coil. fMRI data were acquired using a multiband echo‐planar imaging (EPI) sequence developed by the Center for Magnetic Resonance Research (CMRR) at the University of Minnesota (https://www.cmrr.umn.edu/multiband/). This sequence, incorporating simultaneous multislice (SMS) acceleration, was optimized for blood oxygenation level–dependent (BOLD) contrast to enable high temporal resolution while minimizing slice repetition time and maximizing brain coverage (Setsompop et al. 2012; Xu et al. 2013).

Task‐based fMRI scans were acquired with a 110 × 110 matrix, a spatial resolution of 2 × 2 mm^2^, and a slice thickness of 2 mm with no interslice gap. The imaging parameters were as follows: repetition time (TR) = 1000 ms, echo time (TE) = 30 ms, flip angle (FA) = 60°, field of view (FOV) = 220 mm, and a multiband acceleration factor of 6. Each session lasted approximately 30 min, during which 1800 volumes were acquired with phase encoding in the anterior‐to‐posterior (AP) direction.

To mitigate distortions and signal drop‐out, the protocol incorporated an echo spacing of 620 µs and parallel imaging with GRAPPA (GeneRalized Autocalibrating Partially Parallel Acquisitions) at an acceleration factor of 2. Slice timing was interleaved to reduce motion‐related artifacts. Additionally, field maps were acquired for geometric distortion correction. High‐resolution structural images were also obtained, including T1‐weighted images (0.9 × 0.9 × 0.9 mm^3^, TR = 2700 ms, TE = 3.7 ms, TI = 1090 ms, FA = 9°) using selective water excitation for fat suppression and T2‐weighted images (0.9 × 0.9 × 0.9 mm^3^, TR = 2500 ms, TE = 349 ms).

### 2.2 Statistical Analysis

#### 2.2.1 Behavioral Data Analysis

Both response accuracy and response latency (mean reaction time [RT] of correct responses) were analyzed. To examine the effect of task type (learning vs. control) and stage (1–4) on participants’ performance accuracy in our OLM task, we employed a generalized linear mixed‐effects model (GLMM) with a binomial link function. A GLMM was chosen to account for both fixed and random effects, accommodating the hierarchical structure of the data. The analysis was conducted in R (version 4.3.0) using the glmer function from the lme4 package, applying a logistic linear mixed‐effects model (LMM) to model response accuracy as a binary outcome (correct vs. incorrect responses). Given the positively skewed distribution of RT, we used a GLMM with a gamma distribution and a log‐link function for RT of the corrected responses.

The model included stages (1–4), task type (learning vs. control), their interaction, fMRI session (Session 1 or 2), task version (A or B), and block (six blocks in the learning task and two in the control task) as fixed effects. To account for individual variability, we included a random intercept for participants.

The statistical significance of fixed effects and their interactions was evaluated. To further explore the interaction between task type and stage, we conducted post hoc analyses using estimated marginal means (EMMs) via the emmeans package. EMMs were computed for each combination of task type and stage to examine how response accuracy and RT varied between learning and control tasks across the four stages.

#### 2.2.2 fMRI Data Analysis

fMRI data were analyzed using statistical parametric map software SPM12 (version 7771) and MATLAB (R2021b). Structural T1 images underwent skull stripping, bias correction, and normalization to the MNI template while preserving the original voxel size. Functional BOLD images were preprocessed through realignment, unwarping, and slice timing correction (TR = 1 s, 72 slices). Coregistration of T1 and BOLD images was performed, followed by functional image normalization to MNI space using deformation fields. Lastly, spatial smoothing with a 6 mm FWHM Gaussian kernel was applied to improve the signal‐to‐noise ratio and meet GLM assumptions.

For the first‐level analysis of each participant, GLM was constructed to capture task‐related neural activity. The model included regressors (trial onsets, durations) for each experimental condition (learning and control) across four stages (1–4). These events were modeled as stick functions and convolved with statistical parametric map's (SPM's) canonical hemodynamic response function (HRF) to reflect the delayed and dispersed characteristics of the BOLD response. Data from the two task fMRI sessions per participant were concatenated into a single design matrix. Nuisance regressors, including six motion parameters from realignment and framewise displacement, were added to control for head motion artifacts.

Contrasts of interest were defined to compare the task conditions, with the primary contrasts being the t‐contrast of (learning > control and control > learning). Contrast images were generated for each participant and entered into the second‐level group analysis.

The second‐level analysis employed the participant‐level contrast images using one‐sample *t*‐tests to examine overall task‐related activation patterns. The statistical threshold for initial voxel inclusion was set at (*p* < 0.001, uncorrected), with a family‐wise error (FWE) cluster‐corrected *p* value. The resulting SPMs were overlaid on an MNI anatomical template, and anatomical regions were identified using the Harvard–Oxford cortical and subcortical atlases (https://fsl.fmrib.ox.ac.uk/fsl/fslwiki/). Activation maps were visualized using MRIcroGL software (http://www.mricro.com).

Region of interest (ROI) analysis: To investigate patterns of activity changes across learning stages, we identified ten ROIs based on peak activation clusters showing significant differences between the learning and control tasks. These ROIs were selected using a whole‐brain analysis with an FWE correction (FWEc)‐cluster‐corrected threshold of *p* < 0.001.

We then used the Marsbar toolbox (http://marsbar.sourceforge.net/) (Brett et al. 2002) to extract beta values (parameter estimates) for each participant. The beta values were derived from a 5‐mm sphere centered on the MNI coordinates of each peak activation cluster. This extraction process was conducted separately for each of the four stages of both the learning and control task blocks. This method enabled us to examine how neural activity within these key regions evolves across the different stages of learning and control tasks.

#### 2.2.3 fMRI‐Behavior Correlation

To investigate the relationship between behavioral performance and brain activity during the learning task, we conducted an ROI‐based analysis guided by prior findings from a similar OLM study (Gillis et al. 2016). These ROIs were predefined, independent of our results, based on their established involvement in object–location associations, as per the validated ROI analysis framework (Poldrack 2007). In their comprehensive assessment of neuroimaging evidence supporting the cognitive model of OLM, they investigated the object–location binding process during new versus repeated object–location association (object + location novel > object + location repeated). The resulting activated regions included areas of the ventral visual stream (e.g., the fusiform gyri), the lateral occipital complex (LOC), and medial temporal structures (e.g., the hippocampus and parahippocampus).

In our study, we extracted beta values representing neural activation during the learning task for each subject from these predefined ROIs using the same learning contrast from the whole‐brain first‐level analysis. To assess the relationship between brain activation and performance accuracy, we performed a repeated‐measures correlation analysis using the rmcorr package in R (https://cran.r‐project.org/web/packages/rmcorr/). This method was selected because it effectively accounts for within‐subject associations across repeated measures assessed on multiple occasions (Bakdash and Marusich 2017).

#### 2.2.4 Test–Retest Reliability

To assess the reliability of both behavioral and fMRI results across two sessions with parallel task versions, we conducted an intraclass correlation coefficient (ICC) analysis for both behavioral performance and fMRI activation patterns (Caceres et al. 2009). The ICC is a statistical measure used to assess the reliability of measurements or ratings by evaluating the degree of consistency or agreement among multiple raters or measurements of the same subjects (Koo and Li 2016). The ICC was chosen as a suitable metric for TRR because it accounts for both within‐subject and between‐subject variability, offering a robust measure of consistency over repeated measurements (Weir 2005).

For behavioral accuracy, we calculated the ICC for the mean performance accuracy and latency in the learning task across the stages for each participant in both task sessions (Sessions 1 and 2). ICC estimates and their 95% confidence intervals were computed using the irr package in R, employing a two‐way mixed‐effects model with absolute agreement for single measurements (ICC[3,1]), as per McGraw and Wong (1996). This approach evaluates the reliability of individual scores by accounting for both systematic and random measurement variability, ensuring that absolute agreement between sessions is assessed.

For the consistency of fMRI activation, we used the Python‐based prelimit tool to calculate the ICC based on whole‐brain activation patterns derived from the beta values of the learning task activation (Demidenko et al. 2024). The ICC estimates were computed both inside a whole‐brain mask.

ICC values were interpreted as follows: Values below 0.5 indicated poor reliability, 0.5–0.75 indicated moderate reliability, 0.75–0.9 indicated good reliability, and values above 0.9 indicated excellent reliability (Koo and Li 2016).

## 3 Results

### 3.1 Behavioral Data

**FIGURE 2:**
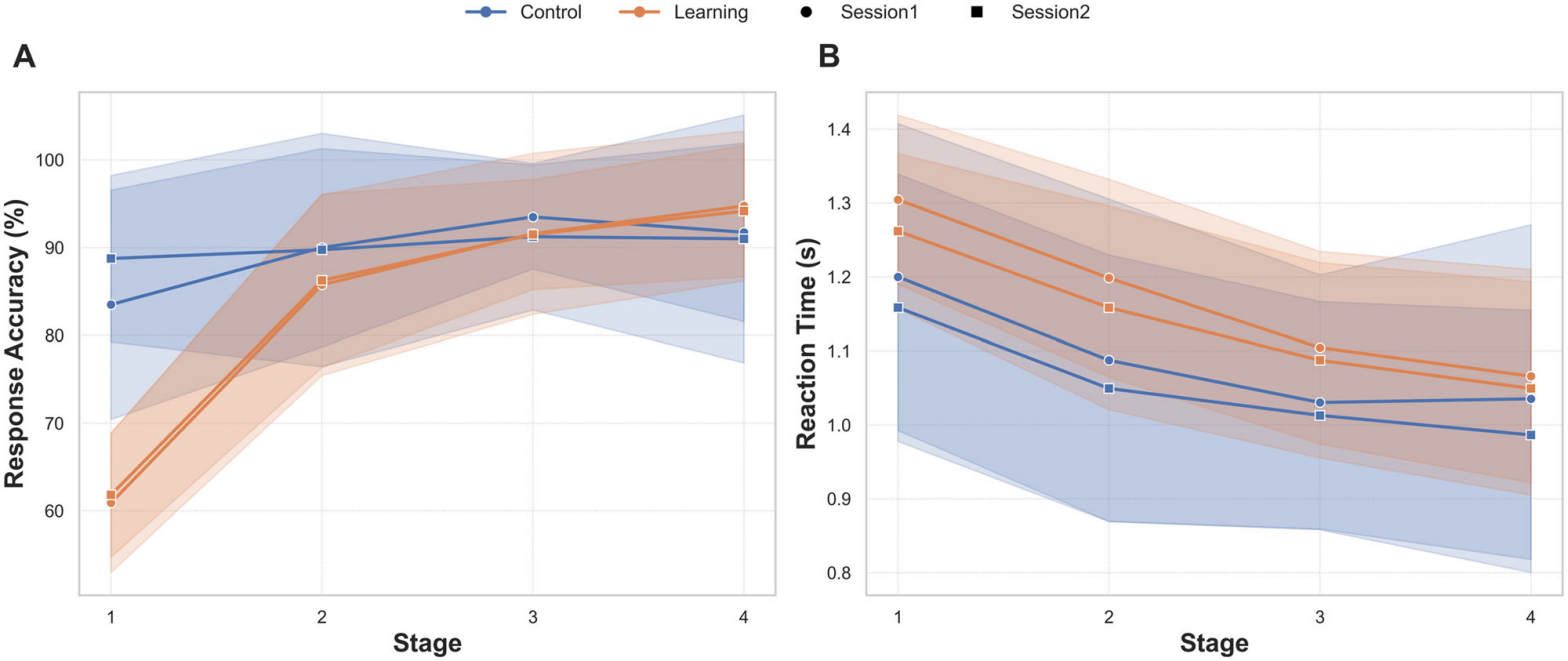
**Performance accuracy (A) and reaction time (B) comparisons for learning vs. control tasks**. (A) Mean response accuracy (%) and (B) reaction time (seconds) across learning stages (1–4) for the learning task (orange) and control task (blue). Data are shown separately for the first fMRI session (circle markers) and the second fMRI session (square markers) for 20 participants. Shaded regions represent standard deviation (SD).

Overall, the results showed a gradual increase in performance accuracy scores and a decrease in RT across the four stages of the learning task. The overall means and patterns of change across the stages are shown in Figure 2.

#### 3.1.1 Response Accuracy

Behavioral data analysis revealed significant improvements in performance accuracy across stages, with distinct trends based on task type (learning vs. control). The GLMM showed a significant effect of stage (estimate = 0.211, SE = 0.054, 95% CI = [0.105, 0.316], *z* = 3.89, *p* < 0.001) and task type (estimate = −2.03, SE = 0.168, 95% CI = [−3.365, −1.706], *z* = −12.11, *p* < 0.001), as well as a significant interaction between stage and task type (estimate = 0.70, SE = 0.06, 95% CI = [0.578, 0.827], *z* = 11.22, *p* < 0.001), indicating that the effect of stage on accuracy differed between the learning and control tasks, with greater improvement in performance observed across stages in the learning condition.

Post hoc pairwise comparisons were conducted for the response accuracy model to explore differences between task types (control vs. learning) across the four stages using EMMs. In Stage 1, accuracy was significantly higher in the control task compared to the learning task (estimate = 1.33, SE = 0.12, *z* = 10.90, *p* < 0.001). In Stage 2, the control task continued to outperform the learning task, although the difference decreased (estimate = 0.63, SE = 0.097, *z* = 6.47, *p* < 0.001). By Stage 3, there was no significant difference between the two tasks (estimate = −0.073, SE = 0.11, *z* = −0.66, *p* = 0.51). In Stage 4, accuracy in the learning task surpassed that of the control task, with a significant negative difference (estimate = −0.77, SE = 0.15, *z* = −5.21, *p* < 0.001).

Other tested fixed effects, including session (*p* = 0.614) and block (*p* = 0.576), were not significant. However, the task version showed a small but significant effect, with participants achieving slightly higher scores during Task B than during Task A (estimate = 0.13, *p* = 0.013).

#### 3.1.2 Reaction Time

The GLMM revealed significant effects of stage and task type on RT. RT decreased across stages (estimate = −0.050, SE = 0.004, *t* = −11.30, *p* < 0.001), suggesting faster responses across stages. Participants in the learning task had slower RTs than those in the control task (estimate = 0.109, SE = 0.014, *t* = 7.49, *p* < 0.001), likely due to the higher cognitive demands of the learning task. The interaction between stage and task type was also significant (estimate = −0.105, SE = 0.005, *t* = −2.01, *p* = 0.044). Session significantly impacted RT, with faster responses in Session 2 (estimate = −0.021, SE = 0.005, *t* = −4.23, *p* < 0.001). Task version (A vs. B) did not affect RT (estimate = 0.009, SE = 0.005, *t* = 1.85, *p* = 0.064).

Post hoc pairwise comparisons showed significant differences in RTs between tasks, with learning trials having longer RTs than control trials at all stages. In Stage 1, RTs were slower in the learning task (estimate = −0.099, SE = 0.010, *z* = −9.86, *p* < 0.0001), with a similar pattern in Stage 2 (estimate = −0.088, SE = 0.006, *z* = −13.56, *p* < 0.0001) and Stage 3 (estimate = −0.078, SE = 0.006, *z* = −12.45, *p* < 0.0001). By Stage 4, the difference decreased but remained significant (estimate = −0.067, SE = 0.009, *z* = −7.08, *p* < 0.0001).

### 3.2 fMRI Data

#### 3.2.1 Whole‐Brain fMRI Analysis

**FIGURE 3:**
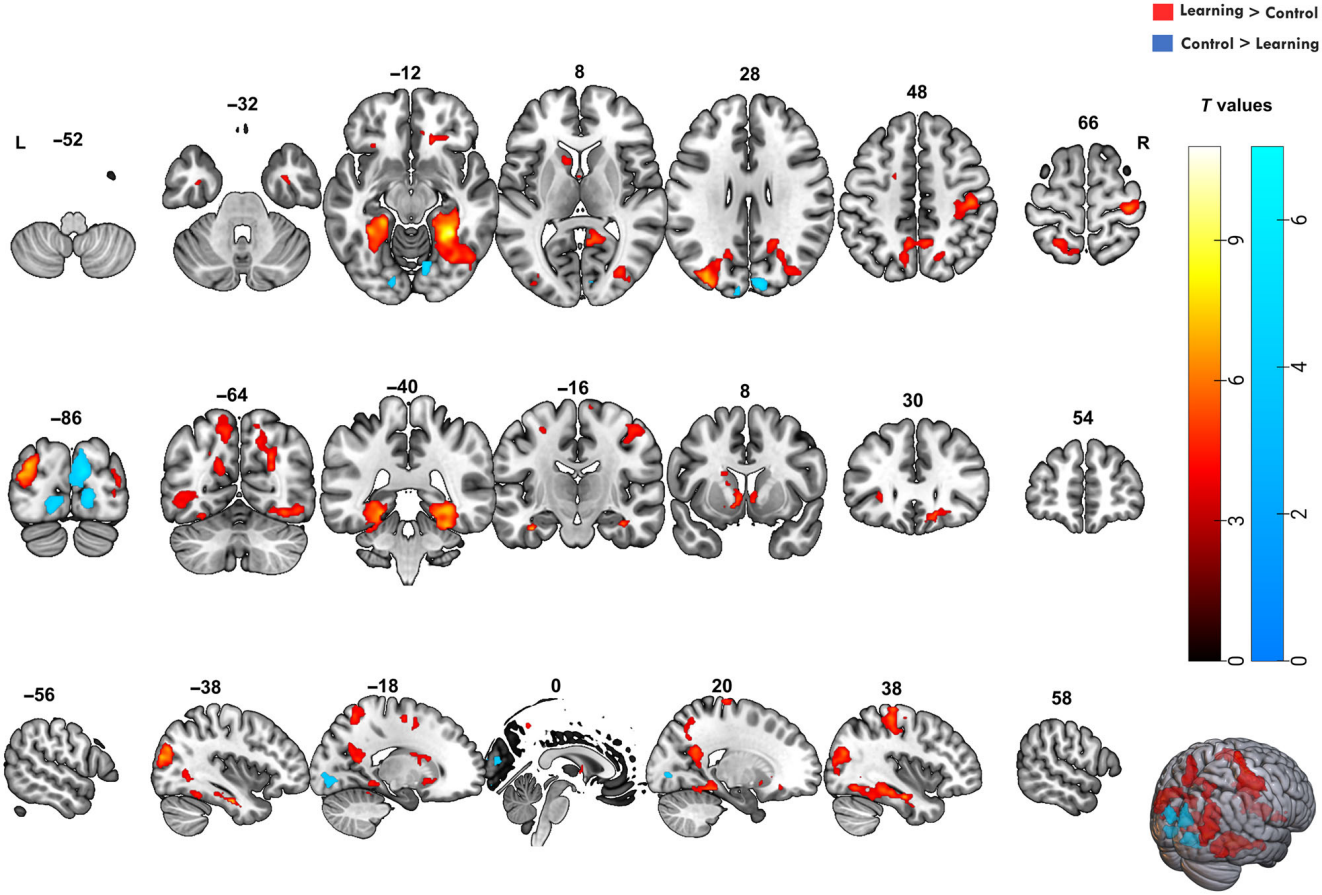
**Brain activation patterns associated with the learning and control tasks**. Statistical parametric maps overlaid on an MNI brain template. Hot color map clusters represent the (learning > control) contrast, and blue clusters represent the (control > learning) contrast for group analysis of the participants (*n* = 20), modeled across two task sessions. The significance threshold was set at *p* < 0.001, with FWE correction applied at the cluster level. The color bar represents *T* values, and brain activations are displayed in axial, coronal, and sagittal views.

For the contrast learning > control, several significant clusters of brain activity were identified. We applied a cluster‐based FWEc with a threshold of *p* < 0.001 and included clusters larger than 125 voxels. Task‐related activations were found mainly in the temporo‐occipital fusiform cortex, hippocampus, precuneus, precentral gyrus, and lateral occipital cortex. The significant clusters and their associated MNI coordinates, *T* values, and voxel sizes are provided in Table 2. Figure 3 displays the SPM overlaid on the MNI brain template.

**TABLE 2:** Significant brain activations for the contrast learning > control.

Harvard–Oxford cortical–subcortical structural atlas	Side	Cluster size (voxels)	*T* values	Peak coordinates (MNI)		
				*X*	*Y*	*Z*
Temporal–occipital fusiform cortex	R	1580	11.03	30	−46	−14
Temporal fusiform cortex, posterior division	R		9.12	30	−36	−22
Right hippocampus	R		7.7	36	−14	−22
Temporal–occipital fusiform cortex	L	1052	9.43	−32	−58	−16
Temporal fusiform cortex, posterior division	L		8.98	−26	−36	−18
Temporal–occipital fusiform cortex	L		8.93	−30	−48	−16
Lateral occipital cortex, superior division	L	998	7.87	−34	−86	26
			7.77	−40	−86	18
			5.34	−26	−72	30
Lateral occipital cortex, superior and inferior division	R	1348	7.42	32	−78	4
			6.59	40	−74	22
			6.42	40	−84	14
Caudate	R	225	7.09	12	20	−4
Accumbens	R		5.73	6	12	−8
Putamen	R		4.69	20	10	−8
Precentral gyrus	R	936	7.08	34	−26	68
Precentral gyrus	R		6.61	16	−24	76
Postcentral gyrus	R		6.42	44	−22	50
Accumbens	L	459	6.61	−8	14	−4
Frontal orbital cortex	R	125	6.02	16	30	−14
Frontal orbital cortex	R		4.34	26	32	−14
Frontal medial cortex	R		3.86	8	38	−14
Precuneus cortex	L	671	5.82	−6	−56	50
Precuneus cortex	R		5.56	8	−54	46
Lateral occipital cortex, superior division	L		5.54	−10	−70	54
Precentral gyrus	L	176	5.68	−16	−10	56
Precentral gyrus	L		5.45	−28	−14	54
Superior frontal gyrus	L		4.68	−20	0	56
Frontal orbital cortex	L	151	5.05	−28	26	−4
Insular cortex	L		4.49	−32	18	−2
Frontal orbital cortex	L		4.2	−32	24	−14
Inferior temporal gyrus, temporo‐occipital part	L	221	4.74	−40	−60	−4
Lateral occipital cortex, inferior division	L		4.43	−48	−64	−6
			4.41	−46	−64	2

*Note*: Several significant clusters of brain activity were identified for the contrast learning > control. A cluster‐based family‐wise error correction (FWEc) was applied, using a threshold of *p* < 0.001 and clusters larger than 125 voxels. The table presents the brain regions identified on the basis of the Harvard–Oxford cortical and subcortical structural atlas, along with the hemisphere (Right, R, or Left, L), the cluster size in voxels, and the *T* values and peak coordinates (in MNI space, *X*, *Y*, *Z*).

##### 3.2.1.1 Temporo‐Occipital Areas

The largest cluster was found in the right temporal–occipital fusiform cortex (1580 voxels, *T* = 11.03, MNI: 30, −46, −14), which extended into the posterior division of the temporal fusiform cortex (*T* = 9.12, MNI: 30, −36, −22) and right hippocampus (*T* = 7.7, MNI: 36, −14, −22). Similarly, a significant cluster was observed on the left side (1052 voxels, *T* = 9.43, MNI: −32, −58, −16), suggesting a bilateral involvement in the learning of object location associations.

##### 3.2.1.2 Hippocampal and Subcortical Regions

In the right hippocampus (*T* = 7.7, MNI: 36, −14, 22), we observed significant activation, aligning with the well‐established role of it in the OLM. Activation was also noted in other subcortical structures, including the caudate (*T* = 7.09, MNI: 12, 20, −4) and accumbens nuclei bilaterally (right: *T* = 5.73, left: *T* = 6.61), which have been linked to learning and reward‐based processing.

##### 3.2.1.3 Precentral and Postcentral Gyri

Additional activity in regions associated with motor execution and somatosensory integration was found, including the precentral gyrus bilaterally (right: 936 voxels, *T* = 7.08, MNI: 34, −26, 68; left: 176 voxels, *T* = 5.68, MNI: −16, −10, 56) and adjacent right postcentral gyrus (right: *T* = 6.42, MNI: 44, −22, 50). This pattern likely reflects the motor demands of the task, such as the use of response grips.

##### 3.2.1.4 Occipital Cortex and Visual Areas

Several regions of the lateral occipital cortex involved in higher order visual–spatial learning were engaged during the task, particularly the superior (left: 998 voxels, *T* = 7.87, MNI: −34, −86, 26; right: 1348 voxels, *T* = 7.42, MNI: 32, −78, 4) and inferior divisions (left: *T* = 4.41, MNI: −46, −64, 2; right: *T* = 6.59, MNI: 40, −74, 22).

##### 3.2.1.5 Frontal and Parietal Areas

Additionally, activations were detected in the frontal orbital cortex bilaterally (right: *T* = 6.02, MNI: 16, 30, −14; left: *T* = 5.05, MNI: −28, 26, −4), which is involved in decision‐making and reward‐related processing, potentially contributing to the learning feedback and memorization components of the task. The precuneus showed bilateral activations (right: *T* = 5.56, MNI: 8, −54, 46; left: *T* = 5.82, MNI: −6, −56, 50), supporting its role in episodic memory encoding and retrieval, especially with previously learned locations.

For the contrast of control > learning, significant activations were only found in regions primarily involved in visual processing, particularly in occipital and lingual areas. Table 3 summarizes the key clusters of activation. For this contrast, significant activations were observed in the cuneal cortex of the right hemisphere (909 voxels, *T* = 6.03, MNI: 12, −86, 22), extending into the lingual gyrus (*T* = 5.94, MNI: 16, −82, −2). Additionally, the right lingual gyrus was active (*T* = 5.26, MNI: 12, −74, −10), which is known to support visual processing and analysis, specifically relating to visual attention and object recognition.

**TABLE 3:** Significant brain activations for the contrast (control > learning).

Harvard–Oxford cortical–subcortical structural atlas	Side	Cluster size (voxels)	*T* values	Peak coordinates (MNI)		
				*X*	*Y*	*Z*
Cuneal cortex	R	909	6.03	12	−86	22
Lingual gyrus	R		5.94	16	−82	−2
Lingual gyrus	R		5.26	12	−74	−10
Lingual gyrus	L	226	4.92	−14	−86	−8
Occipital pole	L		4.22	−18	−90	0

*Note*: Significant clusters of brain activity were identified for the contrast control > learning. A cluster‐based family‐wise error correction (FWEc) was applied with a threshold of *p* < 0.001. The table provides details of the brain regions identified using the Harvard–Oxford cortical and subcortical structural atlas, specifying the hemisphere (Side, R = Right, L = Left), cluster size in voxels, the *T* values, and the peak coordinates (MNI space, *X*, *Y*, *Z*).

In the left hemisphere, a smaller cluster was noted in the lingual gyrus (226 voxels, *T* = 4.92, MNI: −14, −86, −8), with another region activated in the occipital pole (*T* = 4.22, MNI: −18, −90, 0). These regions have been implicated in early‐stage visual processing and contributing to spatial orientation and scene recognition, which were likely engaged by the visual and perceptual nature of the control task.

#### 3.2.2 ROI Analysis

**FIGURE 4:**
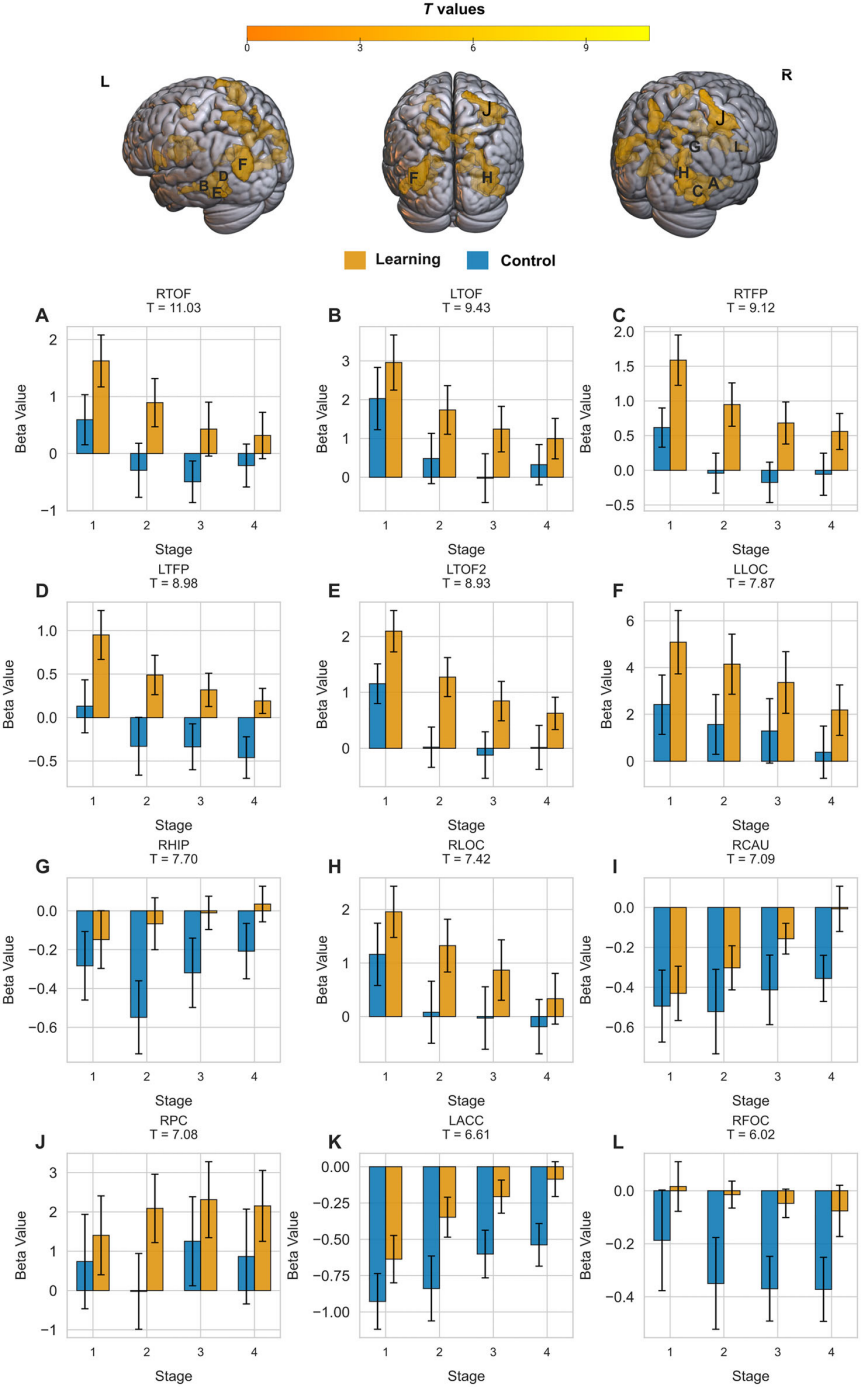
**Task‐related brain activation and parameter estimates across stages in key regions of interest (ROIs)**. The top panel shows brain renderings with significant clusters (cluster‐based FWE‐corrected, *p* < 0.001) from a whole‐brain analysis contrasting the learning task with the control task (learning > control). The bottom panel presents bar plots of average parameter estimates (beta values) for the learning (yellow) and control (blue) tasks across four stages in key ROIs, reflecting task‐related activity. ROIs are labeled with abbreviations with the corresponding *T* values for the learning > control contrast arranged from the highest to the lowest values as follows: (A) RTOF (right temporal occipital fusiform), (B) LTOF (left temporal occipital fusiform), (C) RTFP (right temporal fusiform posterior), (D) LTFP (left temporal fusiform posterior), (E) LTOF2 (left temporal occipital fusiform), (F) LLOC (left lateral occipital cortex), (G) RHIP (right hippocampus), (H) RLOC (right lateral occipital cortex), (I) RCAU (right caudate), (J) RPC (right precentral), (K) LACC (left accumbens), and (L) RFOC (right frontal orbital cortex). The *X*‐axis represents the stage of learning (1–4), the *Y*‐axis represents beta values, and the error bars represent the standard error of the mean. A full list of all significant ROIs is presented in Figure S1.

In most of the ROIs analyzed, as shown in Figure 4, brain activity followed a declining trend, with a gradual reduction in activity as participants progressed through the stages. This pattern suggests that these regions play a critical role in the initial learning and encoding of object locations. However, they become less active as participants consolidate and refine their memory of house locations. The right hippocampus exhibited an opposing pattern of increased activity across the stages of the learning task, along with an inconsistent pattern in the control task, which may support its later involvement in memory consolidation as learning progresses.

#### 3.2.3 FMRI‐Behavioral Correlation

**FIGURE 5:**
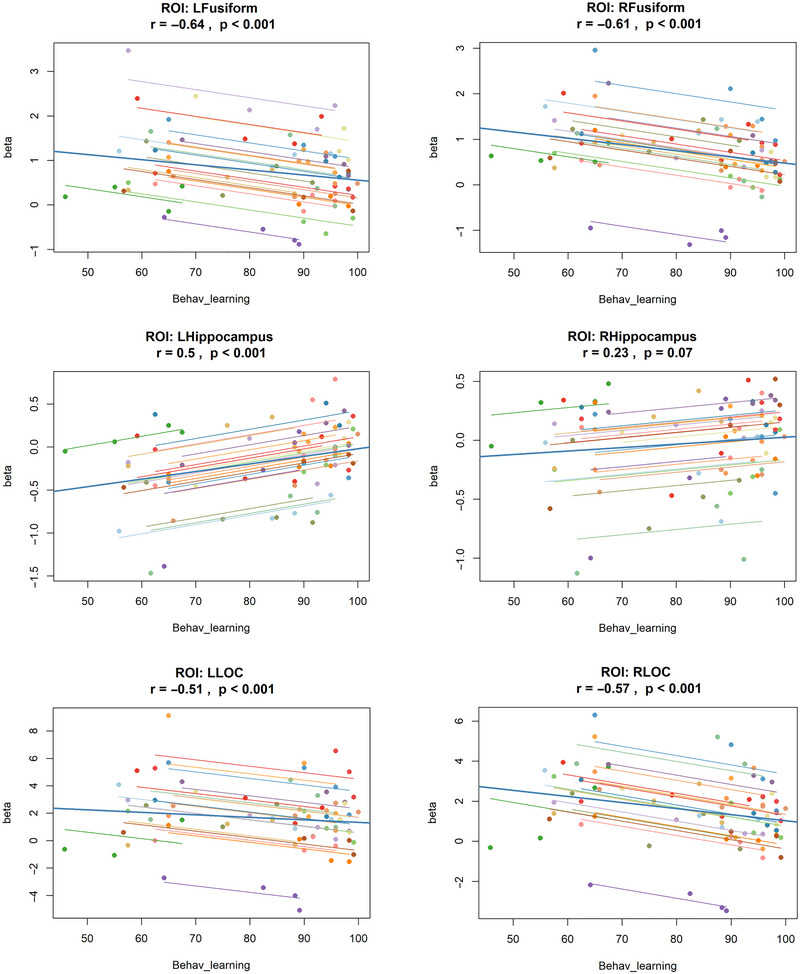
**Repeated‐measures correlations (rmcorr) plots between behavioral learning accuracy and fMRI activation (beta) in the predefined ROIs**: right fusiform (RFusiform), left fusiform gyri (LFusiform), right hippocampus (RHippocampus), left hippocampus (LHippocampus), right lateral occipital complex (RLOC), and left lateral occipital complex (LLOC). Each colored line represents data from an individual participant (*n* = 20) across four stages of learning (four colored dots), with the mean correlation line across participants in blue. Correlation coefficients (*r*) and significance levels (*p* values) are indicated for each ROI.

Figure 5 summarizes the correlation analyses, showing the relationship between neural activation (beta values) and behavioral learning accuracy across the four stages. Significant negative correlations were observed in bilateral LOC, right LOC (*r* = −0.57, *p* < 0.001), and left LOC (*r* = −0.51, *p* < 0.001), as well as in bilateral fusiform gyri; right fusiform (*r* = −0.61, *p* < 0.001) and left fusiform gyrus (*r* = 0.64, *p* < 0.001). In line with their unique activation pattern across the later stages of learning, as shown in the earlier analysis, the left hippocampus also exhibited a significant positive correlation with performance (*r* = 0.50, *p* < 0.001). The right hippocampus also showed a positive trend (*r* = 0.23, *p* = 0.07), but it did not reach statistical significance. These findings are consistent with the previously mentioned behavioral and ROI analysis pattern.

### 3.3 Test–Retest Reliability

#### 3.3.1 Behavioral Results

To assess the TRR of participants’ performance on our OLM task across sessions, an ICC analysis was conducted (Leyland and Groenewegen 2014). A two‐way model (single score with absolute agreement) was applied in R to evaluate the agreement of mean response accuracy scores across both measurement sessions.

The analysis revealed an ICC value of 0.801 (95% CI [0.737, 0.85]), indicating good reliability. This suggests that participants’ accuracy in the OLM task was highly consistent across sessions. The *F*‐test for the ICC was significant (*F* (159,159) = 9, *p* < 0.001). For the RT, the resulting ICC was 0.705 (95% CI [0.613, 0.777]), indicating moderate reliability across both sessions. This suggests that participants’ RTs demonstrated reasonable consistency between sessions, though with slightly lower reliability than accuracy scores. The ICC was statistically significant, *F* (159,133) = 6, *p* < 0.001. These findings support the stability of participants’ performance over time and underscore the reliability of our OLM task.

#### 3.3.2 fMRI Results

**FIGURE 6:**
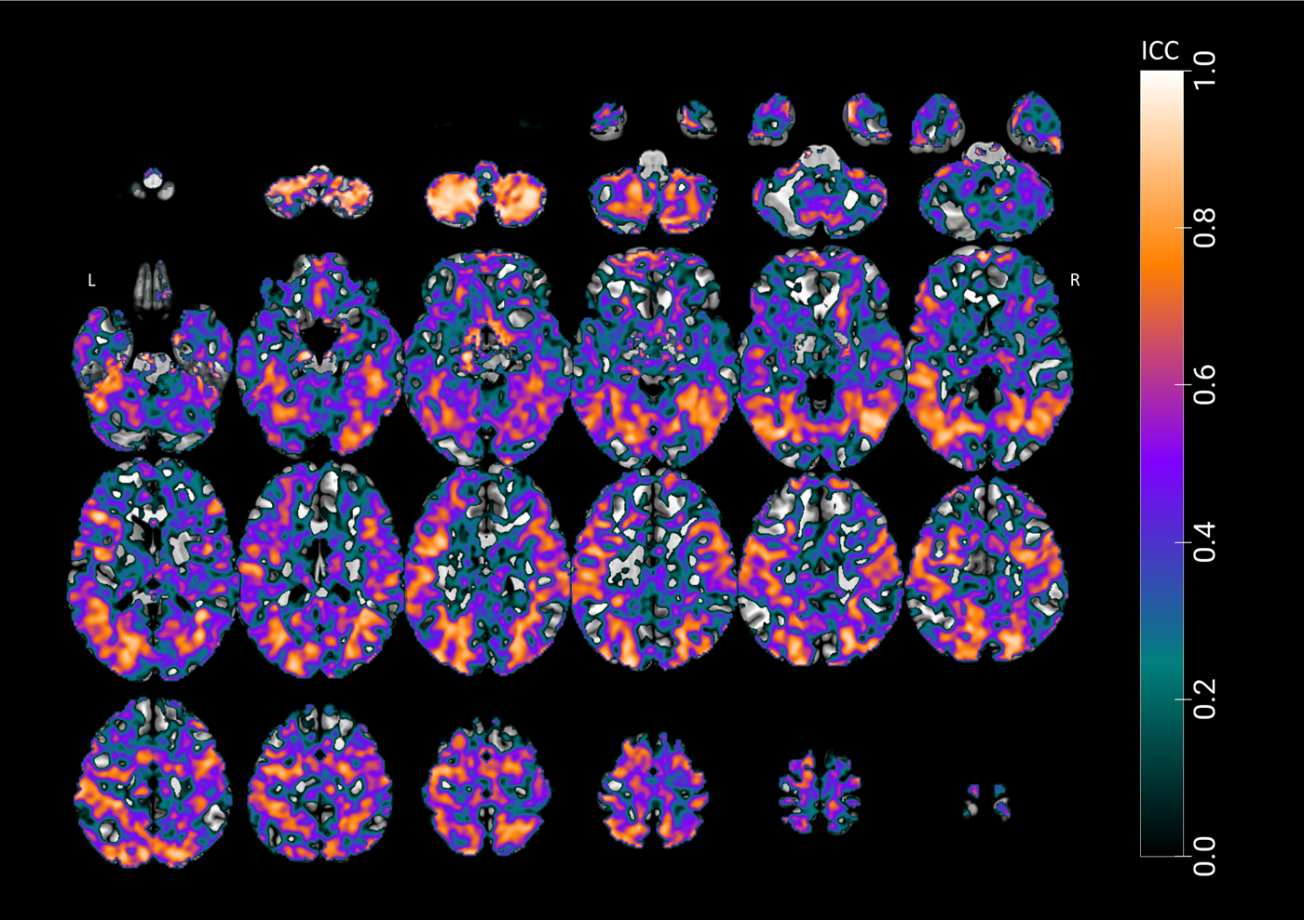
**Intraclass correlation coefficient (ICC) maps for fMRI activations during the learning task across two task sessions**. This figure presents whole‐brain ICC maps overlaid on an MNI template, illustrating the distribution of ICC values across various brain regions. The accompanying color bar indicates ICC values ranging from 0 to 1, where warm colors (orange to yellow) signify high ICC values, and cool colors (green to blue) denote low ICC values.

To assess the TRR of fMRI activations during the learning task, ICC maps were generated for task‐related activity, contrasting learning with control tasks across two sessions. The resulting ICC map, presented in Figure 6, reveals a pattern of moderate to excellent reliability in several key regions associated with learning and memory processes. Bilateral regions, including the fusiform gyri, middle temporal gyri, and lateral occipital cortices, demonstrated moderate‐to‐good reliability across both sessions.

Notably, the right hemisphere exhibited particularly high ICC values in several areas. The right middle temporal gyrus showed excellent reliability across its anterior division (MNI: 42, −55, 13; ICC = 0.902), posterior division (MNI: 41, −55, 12; ICC = 0.910), and temporo‐occipital part (MNI: 42, −54, 12; ICC = 0.918). Similarly, the right lateral occipital cortex demonstrated good‐to‐excellent reliability in both its superior division (MNI: 25, −76, 24; ICC = 0.870) and inferior division (MNI: 47, −65, 0.5; ICC = 0.922). The right temporal–occipital fusiform cortex also showed good reliability (MNI: 30, −52, −8; ICC = 0.845).

Consistent patterns of reliability were observed in the left hemisphere. The left lateral occipital cortex exhibited good ICC values (MNI: −41, −79, −2; ICC = 0.803) alongside other regions, including the left middle temporal gyrus temporo‐occipital part (MNI: −40, −64, 1.9; ICC = 0.843), superior temporal gyrus (MNI: −60, −33, 6; ICC = 0.804), lateral occipital cortex (MNI: −39, −75, 0; ICC = 0.857), and temporal fusiform cortex (MNI: −20, −72, −6; ICC = 0.824). The precuneus cortex has also shown moderate reliability (MNI: −6, −56, 50; ICC = 0.655).

Conversely, lower ICC values, shown in blue on the maps, were observed in regions, such as bilateral frontal regions, ventral temporal cortices, and white matter. These areas, unrelated to task‐specific demands, likely reflect noise or non‐specific activation patterns. These findings support the experimental design and the reliability of fMRI activations elicited during the OLM task.

## 4 Discussion

This study utilized a novel fMRI adaptation of an established OLM paradigm to investigate behavioral and neural correlates of visual–spatial learning in 20 healthy young‐ to middle‐aged adults. The primary objectives were to identify potential cortical targets for tDCS and to assess the TRR of behavioral and neural outcomes. Findings revealed several cortical regions implicated in OLM, including the occipito‐temporal regions, precuneus, and fusiform gyri, which may serve as viable targets for tDCS. Additionally, both behavioral measures and task‐active brain regions demonstrated moderate to excellent TRR, supporting the paradigm's reliability for future tDCS applications.

Behaviorally, the OLM task elicited significant learning across four stages of feedback‐based associative learning, distinguishing it from the control condition, which lacked explicit memory demands. Neuroimaging findings revealed a right‐dominant activation pattern in temporo‐occipital and medial temporal regions, including the fusiform gyri and right hippocampus, consistent with the neurocognitive model of OLM (Postma et al. 2004). These results confirm the engagement of lateral temporo‐occipital cortices, which may serve as accessible tDCS targets. In contrast, activation in the control task was limited to primary visual and medial occipital areas, which are associated with low‐level visual processing rather than higher order memory functions (Grill‐Spector and Malach 2004; Palejwala et al. 2021).

Several studies have investigated tDCS for OLM enhancement, yet findings remain inconsistent (Flöel et al. 2012; England et al. 2015; Külzow et al. 2017; Prehn et al. 2017; Bjekić et al. 2019; de Sousa et al. 2020; Fromm et al. 2024). Most of these studies targeted parietal regions—specifically T6 (Flöel et al. 2012; Külzow et al. 2017; Antonenko et al. 2018; de Sousa et al. 2020), CP6 (Fromm et al. 2024), or P3/P4 (England et al. 2015; Bjekić et al. 2019)—based on the EEG 10–20 system. However, these selections were informed by limited neuroimaging evidence and often relied on paradigms that differed methodologically from tDCS studies, which may have contributed to inconsistent outcomes.

To address these limitations, we adapted an established OLM paradigm (Flöel et al. 2012; Külzow et al. 2014) for fMRI, enabling direct investigation of its neural correlates rather than relying on prior neuroimaging studies with differing methodologies. Unlike prior studies emphasizing parietal involvement, our findings revealed stronger engagement of temporo‐occipital regions, including the hippocampus, fusiform gyri, and lateral temporo‐occipital areas. Although the hippocampus is less accessible to non‐invasive stimulation (Toth et al. 2024), the superficial location of temporo‐occipital cortices makes them promising tDCS targets (Barbieri et al. 2016; Kikuchi et al. 2017; Brunyé et al. 2017).

The occipito‐temporal cortices, including the LOC, have previously been implicated in both object recognition and object–location binding, particularly in the right hemisphere (Grill‐Spector et al. 2001; Gillis et al. 2016). Although tDCS targeting of this region has been studied for enhancing object memory (van Meel et al. 2016; Cacciamani et al. 2024), its potential for improving OLM remains underexplored. The anatomical accessibility of these regions supports focal tDCS applications with minimal current spread (Datta et al. 2009; Alam et al. 2016).

Among the most active regions were the fusiform gyri, which play a crucial role in high‐level visual processing and visuospatial memory (Petersson et al. 2001; Weiner and Zilles 2015). As part of the ventral visual stream, these structures support both object‐only and object‐in‐location retrieval (Ross and Slotnick 2008; Gillis et al. 2016). Although conventional tDCS protocols targeting a single fusiform gyrus (e.g., P10 in the EEG system) have been explored for enhancing working memory (Brunyé et al. 2017), its anatomical depth poses challenges for focal neuromodulation, as conventional tDCS may induce diffuse and non‐specific current flow (Reinhart and Woodman 2015).

The precuneus also exhibited bilateral activation, reinforcing its role in visual perception and spatial memory, particularly in encoding visuospatial representations into long‐term memory (Schott et al. 2018; Cohen et al. 2019) and in retrieving previously learned locations (Bogler et al. 2024). It is part of larger scale brain networks, such as the default mode network, and is strongly connected to the hippocampus (Sestieri et al. 2011; Lefaucheur et al. 2020). Prior research has demonstrated that stimulation of the precuneus enhances episodic memory retrieval in both healthy individuals (Bonnì et al. 2015; Benussi et al. 2022) and patients with early Alzheimer's disease (Koch et al. 2018), making it a viable target for OLM modulation.

We also observed significant activation in the precentral gyrus during the task. The precentral gyrus, corresponding to the primary motor cortex (M1), is primarily involved in motor planning and execution (Hanakawa et al. 2008). An fMRI study examining individual task periods during object and location memory found that activation in the precentral and postcentral gyri was associated with motor responses, specifically button presses during the response period (Passaro et al. 2013). Similarly, another study incorporating both motor and spatial memory components demonstrated that precentral gyrus activation typically occurs during motor‐related paradigms rather than during spatial memory‐specific processing (Simon et al. 2002). Furthermore, tDCS targeting M1 has typically been employed to enhance motor learning rather than memory performance (Ehsani et al. 2025). Thus, the precentral activity observed here is best interpreted as reflecting motor execution demands rather than specific OLM processing. Additional regions activated by our task included the frontal orbital cortices, potentially reflecting feedback‐based learning and decision‐making (Wallis 2007). However, their deep location renders them less accessible to non‐invasive brain stimulation (Rao et al. 2018).

Most activated regions exhibited bilateral engagement with a right‐hemispheric predominance, consistent with previous OLM research (Postma et al. 2008). Notably, some recent lesion and neuroimaging studies have not confirmed a consistent lateralization pattern, as hemispheric dominance may vary with task design (e.g., categorical vs. coordinate or egocentric vs. allocentric presentations) (Zimmermann and Eschen 2017). Our findings, on the basis of our paradigm, support a right‐hemispheric preference for OLM, aligning with previous right‐lateralized tDCS protocols (Flöel et al. 2012; Antonenko et al. 2018).

Across learning stages, fMRI activation peaked early in the task and declined over time, consistent with repetition suppression effects (Breitenstein et al. 2005; Engstrom et al. 2013). This reduction in activation, coupled with negative correlations between behavioral accuracy and activation, suggests increased neural efficiency as learning progresses (Vartanian et al. 2022). However, it may also indicate a decline in task engagement over time. Future tDCS studies may target these regions to enhance early learning or sustain engagement when activity diminishes (Falcone et al. 2018), potentially accelerating learning or preventing plateau effects (Sliwinska et al. 2017; Framorando et al. 2021).

Interestingly, right hippocampal activation increased during later learning stages, suggesting a shift from encoding to retrieval and integration (Zhan et al. 2018). However, other studies have reported hippocampal repetition suppression during learning (Vannini et al. 2012; Guo et al. 2021), reflecting dynamic hippocampal involvement influenced by subregional specificity, task demands, or methodological differences (Staresina and Davachi 2009; Davachi and DuBrow 2015; Schlichting et al. 2015). Future research should explore these dynamics to optimize tDCS timing, given its dependence on neural activity states (Kronberg et al. 2017; Ney et al. 2021), which underscores the potential for phase‐specific stimulation to enhance learning outcomes.

This study also assessed the TRR of behavioral and neuroimaging measures, addressing the variability often reported in tDCS‐fMRI research (Esmaeilpour et al. 2020; Meinzer et al. 2024). In fMRI studies, TRR estimates are rarely reported, and when available, they often indicate poor reliability (B. L. Elliott, McClure, et al. 2020; Compère et al. 2021). Similarly, tDCS effects often show low reliability across sessions (Dyke et al. 2016; Wörsching et al. 2017). This variability in brain activation and tDCS outcomes highlights the importance of assessing TRR before conducting interventional studies to ensure reliable conclusions about efficacy. Using ICCs, we found good reliability for accuracy measures, supporting their use as a primary behavioral outcome. Additionally, we observed moderate to excellent reproducibility of fMRI activation in key regions, which supports their use in crossover tDCS studies.

A potential limitation of this study is the small sample size, which may affect the generalizability of our TRR findings, as small samples can inflate reliability estimates (Kriegeskorte et al. 2010). However, the use of a well‐controlled block design and ICC, which accounts for both within‐ and between‐subject variability, reduces the risk of spurious results. Replication in larger cohorts is recommended to confirm the stability of these effects.

Although the overall accuracy and RT patterns of the OLM task demonstrated a clear learning curve and strong reliability across both testing sessions, unexpectedly low performance was observed in the control task, particularly during the first fMRI session at the first stage (mean accuracy [SD] = 83.5% [33.5]). This lower‐than‐expected performance may be attributed to participants’ unfamiliarity with the MRI environment and the use of the MRI‐compatible response grips. To reduce initial performance variability and to improve response compliance, we recommend incorporating a brief familiarization session using the same response grips prior to scanning. Additionally, behavioral data showed no significant differences between the final two learning stages, suggesting a ceiling effect that may have masked further modulation in brain activation. Nevertheless, the learning curve remained well‐defined across the first three stages, indicating effective task engagement. For subsequent studies, task difficulty might be slightly increased to prevent ceiling effects and to maintain participant engagement across all stages (Nawaz et al. 2020). Another limitation of the present study is its reliance solely on fMRI. This choice was driven by the high spatial resolution offered by fMRI, which is particularly relevant for identifying task‐specific neural activations while still providing adequate temporal resolution for studying cognitive processes. However, future research could benefit from integrating complementary neuroimaging modalities, such as PET or MEG, to further validate and extend our findings and to provide a more comprehensive understanding of the underlying neural mechanisms.

## 5 Conclusion

In conclusion, this study supports the utility of an fMRI‐compatible OLM task for investigating the effects of tDCS on memory enhancement. The observed learning curve and consistent activation of regions such as lateral occipito‐temporal cortices, fusiform gyri, and precuneus emphasize their relevance to OLM. Additionally, the stability of both behavioral and functional measures across two testing sessions supports their reliability in crossover interventional studies.

On the basis of the current findings, the most viable cortical targets for tDCS are the lateral occipito‐temporal regions, including the LOC. These regions demonstrated robust activation during object–location learning and are anatomically superficial enough to allow for effective stimulation using focal tDCS protocols.

The precuneus and fusiform gyri also emerged as promising targets, given their consistent engagement during spatial memory encoding and their potential accessibility for non‐invasive DC stimulation, at least with non‐focal tDCS setups (Brunyé et al. 2017). However, the medial location of these regions poses a challenge for focal current delivery. Similarly, deeper structures such as the hippocampus and orbitofrontal cortex, despite their well‐established roles in memory processing, are not accessible with either focal or non‐focal tDCS due to their anatomical depth. Nonetheless, these regions may be indirectly modulated through stimulation of cortical hubs within their respective functional networks using tDCS or transcranial magnetic stimulation (TMS; Wang et al. 2014). Additionally, future studies may consider exploring emerging techniques, such as temporal interference stimulation (TIS), which show potential for directly targeting deep brain structures like the hippocampus (Violante et al. 2023).

## Author Contributions

**Mohamed Abdelmotaleb**: formal analysis, methodology, project administration, validation, visualization, writing – original draft. **Filip Niemann**: data curation, project administration, software, validation, writing – review and editing. **Harun Kocataş**: data curation, formal analysis, investigation, project administration. **Leonardo M. Caisachana Guevara**: data curation, formal analysis, investigation, project administration. **Alireza Shahbabaie**: investigation, methodology, project administration, validation. **Robert Malinowski**: data curation, resources, software. **Steffen Riemann**: data curation, resources, software, validation. **Anna Elisabeth Fromm**: data curation, software, visualization. **Dayana Hayek**: project administration, validation, visualization. **Daria Antonenko**: conceptualization, funding acquisition, methodology, project administration, validation. **Marcus Meinzer**: conceptualization, funding acquisition, methodology, project administration, supervision, visualization, writing – original draft, writing – review and editing. **Agnes Flöel**: conceptualization, funding acquisition, methodology, project administration, resources, supervision, visualization, writing – original draft, writing – review and editing.

## Ethics Statement

The research was conducted at the University Medicine Greifswald and approved by the medical ethics committee (Registry number BB015/22). The study followed the guidelines of the Declaration of Helsinki, and participants provided written informed consent before inclusion.

## Conflicts of Interest

The authors declare no conflicts of interest.

## Peer Review

The peer review history for this article is available at https://publons.com/publon/10.1002/brb3.70658

## Supporting information




**Supporting Fig.1**: Task‐related brain activation across all significant regions of interest (ROIs) across stages.

## Data Availability

This study was pre‐registered on the Open Science Framework (OSF). The pre‐registration protocol is available under OSF Registries (https://osf.io/t37u2). The data that support the findings of this study are available upon request from the corresponding author. The data are not publicly available due to privacy and ethical restrictions related to the study's participants.
